# Meta-analysis of Niaoduqing Granules for uremic pruritus in Chinese hemodialysis patients: efficacy and safety evidence from RCTs

**DOI:** 10.3389/fmed.2025.1622677

**Published:** 2025-11-25

**Authors:** Xue Cao, Xiaofen Ma

**Affiliations:** Blood Purification Center, Zhejiang Hospital, Hangzhou, Zhejiang, China

**Keywords:** Niaoduqing Granules, uremic pruritus and hemodialysis, meta-analysis, chronic kidney disease, pruritus

## Abstract

**Background:**

Uremic pruritus, a common complication in hemodialysis populations, adversely affects quality of life owing to multifactorial etiology. Emerging evidence from traditional Chinese medicine highlights Niaoduqing Granules as a candidate intervention for symptom mitigation. To assess its therapeutic potential, a retrospective meta-analysis was performed to evaluate efficacy and safety profiles in hemodialysis cohorts. These findings aim to inform evidence-based decisions regarding its clinical applicability.

**Methods:**

A systematic literature search was conducted across four international biomedical databases (e.g., PubMed, Embase, Web of Science, ClinicalTrials.gov) and three Chinese scholarly platforms (CNKI, Wanfang, CQVIP), with the search window extending through March 2025. We systematically reviewed all available randomized controlled trials (RCTs) investigating the effects of Niaoduqing Granules on uremic pruritus in hemodialysis populations.

**Results:**

The final analysis incorporated 14 qualifying studies involving 1,232 participants. Hemodialysis patients receiving Niaoduqing Granules demonstrated a significant reduction in pruritus scores compared to the control group (Visual Analogue Scale: WMD = −2.151; 95% CIs = −2.372 to −1.930; *p* < 0.001. 5D-itch Scale: WMD = −7.642; 95% CIs = −14.042 to −1.243; *p* = 0.019). Demonstrates high therapeutic efficacy with a favorable safety profile. This meta-analysis demonstrated clinically meaningful efficacy favoring the Niaoduqing Granule group (RR = 1.249; 95% CIs = 1.169–1.335; *p* < 0.001), with safety analyses across three trials revealing comparable adverse event rates (R = 1.303; 95% CIs = 0.589–2.884; *p* = 0.514; *I*^2^ = 0.0%). In addition, in the Niaoduqing Granules group, significant intergroup differences were observed in serum levels of parathyroid hormone, phosphorus, creatinine, blood urea nitrogen, β2-microglobulin, high-sensitivity C-reactive protein, tumor necrosis factor-α, and interleukin-6, all demonstrating statistical significance.

**Conclusion:**

Niaoduqing Granules demonstrated efficacy in reducing pruritus severity among hemodialysis patients without increasing drug-related adverse events, while also showing concomitant reductions in inflammatory biomarkers. However, limitations in sample size necessitate further large-scale studies to validate these preliminary findings.

## Introduction

Chronic kidney disease (CKD) has become a significant global public health challenge (1). Patients with end-stage renal disease (ESRD) rely on renal replacement therapies such as hemodialysis to sustain life (2). However, hemodialysis patients often experience multiple complications, among which uremic pruritus affects 40–70% of individuals, severely impairing quality of life and correlating with depression, sleep disorders, and increased mortality (3–5). The pathophysiology of pruritus is complex, involving interactions among uremic toxin accumulation, chronic inflammation, neuronal sensitization, and mineral metabolism disorders (6). Current therapies (e.g., antihistamines, gabapentinoids) demonstrate limited efficacy and carry side effects (7), highlighting the urgent need for safer and more effective interventions. Niaoduqing Granules have been used clinically for more than 20 years and their mechanism of action has been comprehensively investigated, comprises *Rheum palmatum*, Astragalus membranaceus, *Morus alba* bark, and other medicinal herbs (8, 9). It is designed to promote bowel excretion to remove turbid toxins and strengthen the spleen to resolve dampness. Clinically, it is widely used to delay CKD progression and reduce serum creatinine and urea nitrogen levels (10, 11). Recent studies suggest, the adjunctive use of Niaoduqing Granules with conventional hemodialysis therapy significantly reduces key biochemical parameters, decrease uremic toxin accumulation, improve renal function indicators, and alleviate the severity of skin pruritus in patients (12–17). For example, Guo Xuewen et al. investigated the efficacy of Niaoduqing Granules in alleviating skin pruritus and removing uremic toxins in hemodialysis patients. The study concluded that Niaoduqing Granules can effectively eliminate blood toxins, regulate calcium-phosphorus metabolism, and improve skin itching in patients undergoing hemodialysis therapy (18). Nevertheless, the components and therapeutic efficacy of Niaoduqing Granules raise notable limitations and concerns in hemodialysis patients, lacking systematic evidence to confirm its efficacy. This meta-analysis aims to comprehensively evaluate the therapeutic effects of Niaoduqing Granules on pruritus in hemodialysis patients, providing evidence-based insights for clinical decision-making.

## Materials and methods

This study adhered to the PRISMA guidelines for systematic reviews and meta-analyses.

### Ethical review

The research methodology utilized exclusively pre-existing scholarly data, thereby eliminating the requirement for institutional review board authorization or individual participant agreements.

### Literature search

Two investigators working independently executed a comprehensive literature retrieval strategy, examining four major international databases (including PubMed, Embase, Web of Science, and ClinicalTrials.gov) alongside three prominent Chinese academic repositories (CKNI, Wanfang, and CQVIP), with the search window extending through March 31, 2025. Search for randomized controlled trials (RCTs) on the use of Niaoduqing Granules in managing uremic pruritus among hemodialysis patients. The search strategy was consisted of free word and MeSH terms, which included: Niaoduqing Granules, uremic pruritus and hemodialysis. Manual searches were conducted through the reference lists of included studies and existing meta-analyses to identify potentially eligible randomized controlled trials (RCTs).

### Inclusion and exclusion criteria

Studies were incorporated into the analysis according to the following criteria in line with the PICOS framework: (I) Participants: patients presenting with hemodialysis-dependent uremic pruritus; (II) Interventions: Hemodialysis patients with uremic pruritus treated with Niaoduqing Granules versus those not treated with Niaoduqing Granules; (III) Comparisons: comparison of treatment safety and pruritus improvement between hemodialysis patients receiving versus not receiving Niaoduqing Granules; (IV) Outcome measures: studies needed to report one or more of the following endpoints—visual analogue scale, 5D-itch scale, effective rate, adverse drug reaction, serum calcium, serum parathyroid hormone, serum phosphorus, serum intact parathyroid hormone, serum creatinine, blood urea nitrogen, uric acid, β2-microglobulin, high-sensitivity c-reactive protein, tumor necrosis factor-α, and interleukin-6; (V) Study design: eligible studies were limited to official, reported, and full-text randomized controlled trials (RCTs).

The exclusion criteria were as follows: (I) studies that did not meet the inclusion criteria, (II) articles in the form of editorials, conference proceedings, commentary articles, reviews, clinical conference reports, and abstracts, (III) studies involving animals, and (IV) duplicate reports from the same database.

### Data extraction

Two independent reviewers performed study selection and data extraction. Discrepancies were resolved through third-author consultation and consensus. Extracted data from eligible studies included: Two independent reviewers performed study selection and data extraction. Discrepancies were resolved through third-author consultation and consensus. Extracted data from eligible studies included: Furthermore, the outcome data were also collected, which comprised: the assessment of visual analogue scale, 5D-itch scale, effective rate, adverse drug reaction, additionally, changes in serum calcium, serum parathyroid hormone, serum phosphorus, serum intact parathyroid hormone, serum creatinine, blood urea nitrogen, uric acid, β2-microglobulin, high-sensitivity c-reactive protein, tumor necrosis factor-α, and interleukin-6, were analyzed following Niaoduqing Granules administration.

### Quality assessment

We used the Cochrane Collaboration tool to evaluate the quality of all included RCTs. More specifically, this study used a ‘risk of bias’ table to show the risk of bias in each trial, including factors like allocation concealment, blinding of outcome assessors, incomplete outcome data, selective outcome reporting, random sequence generation, and other possible sources of bias. Two reviewers independently assessed bias risk (high/unclear/low), with disagreements resolved by consensus or third reviewer involvement. Most RCTs demonstrated moderate quality.

### Statistical analysis

Statistical analysis utilized Stata, assessing heterogeneity with *I*^2^ statistic (<50% threshold). Random-effects meta-analysis followed Cochrane guidelines. For continuous outcomes, such as visual analogue scale, 5D-itch scale, serum calcium, serum parathyroid hormone, serum phosphorus, serum intact parathyroid hormone, serum creatinine, blood urea nitrogen, uric acid, β2-microglobulin, high-sensitivity c-reactive protein, tumor necrosis factor-α, and interleukin-6, the results were presented as weighted mean differences (WMD) with 95% confidence intervals (CIs). Categorical outcomes, including the overall effectiveness rate and adverse drug reaction, were assessed using risk ratio (RR) with 95% CIs. Statistical significance was determined when the *p*-value was less than 0.05.

## Results

### Search results

**Figure 1:**
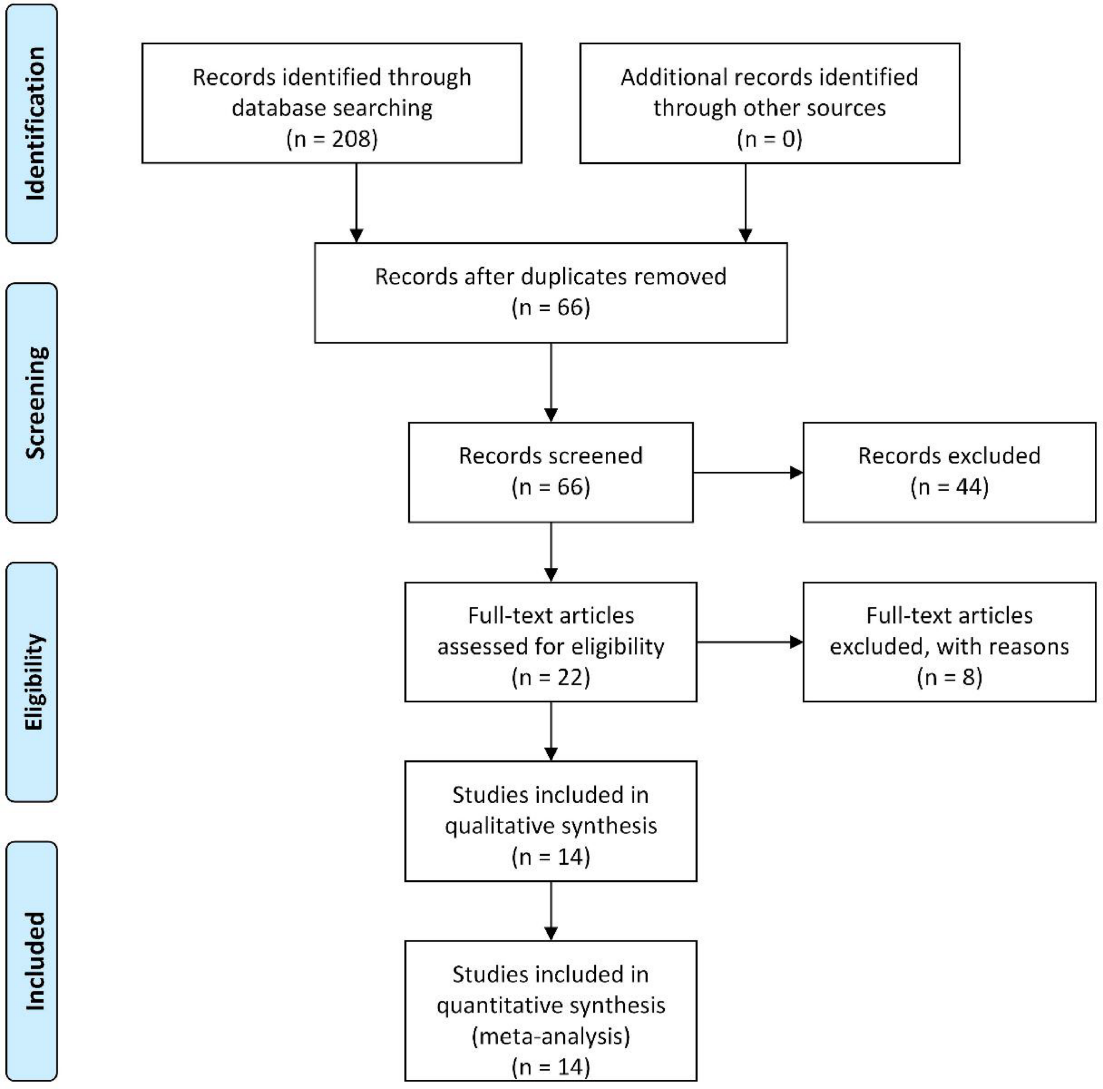
The depicted screening study process.

Initial database searches and supplementary sources yielded 208 potentially relevant articles. Following the import of search results into Endnote software, 142 duplicates were systematically removed. Subsequent title/abstract screening excluded 44 studies deemed irrelevant to the research objectives. The remaining 22 articles underwent full-text assessment, during which rigorous application of predefined inclusion/exclusion criteria and evaluation of data completeness were implemented. Ultimately, 14 randomized controlled trials (RCTs) met the inclusion criteria and were included in the meta-analysis (12–25). The comprehensive selection workflow is presented in Figure 1.

### Study characteristics and quality assessment

The characteristics and demographic data of the patients included in the 14 trials involving 1,232 patients conducted in China are presented in Table 1. All selected studies were RCTs published between 2012 and 2023. Among the 1,232 subjects, all were uremic patients undergoing hemodialysis, all of whom exhibited uremic pruritus of differing severity levels. Of these, 618 patients received Niaoduqing Granule, while 614 did not. Across the 14 included studies, 11 used 5 g orally four times daily (12, 13, 15, 16, 19, 20, 22–25). The remaining 3 studies used alternative regimens: one with 5 g/100 mL twice daily (17), one with 2.5 g/100 mL twice daily (14), and one via enema (15 g/150 mL twice daily) (21). The duration of Niaoduqing Granule treatment varied from 4 weeks to 72 weeks. Of these studies, 10 studies provided information on effective rate (13, 15, 16, 18–24), and 3 studies provided information on adverse drug reactions (12, 20, 25). Of note, there are 10 RCTs that reported the assessment of the severity of pruritus (13, 15–17, 19–24). Further details are provided in Table 1.

**Table 1:** Baseline characteristics of the included studies.

Authors	Year	Sample		Study design	Effective rate		ADR		Treatment method	PSA	Period
		Exper	Control		Exper	Control	Exper	Control			
Yu et al.	2017	65	63	RCT	49/65	36/63	NA	NA	Oral, 5 g, 4 times/d	VAS	12 W
Sun et al.	2018	54	54	RCT	NA	NA	NA	NA	Oral, 5 g/100 mL, 2 times/d	VAS	12 M
Tan et al.	2018	40	40	RCT	38/40	31/40	NA	NA	Oral, 5 g, 4 times/d	NA	12 W
Cao et al.	2019	40	40	RCT	38/40	32/40	NA	NA	Oral, 5 g, 4 times/d	NRS	2 M
Li et al.	2019	52	50	RCT	39/52	28/50	NA	NA	Oral, 5 g, 4 times/d	VAS	12 W
Xi et al.	2021	58	58	RCT	56/58	45/58	NA	NA	Oral, 5 g/150 mL, 4 times/d	VAS	12 M
Zhang et al.	2023	60	60	RCT	NA	NA	5/60	4/60	Oral, 5 g, 4 times/d	VAS + SF-36	12 W
Yang et al.	2016	21	21	RCT	NA	NA	NA	NA	Oral,2.5 g/100 mL, 2 times/d	NA	1 M
Chen et al.	2020	50	50	RCT	42/50	33/50	6/50	4/50	Oral, 5 g, 4 times/d	VAS + 5DIS + DLQI	12 W
Sun et al.	2020	64	64	RCT	NA	NA	2/64	2/64	Oral, 5 g, 4 times/d	5-DIS	12 W
Gao et al.	2012	16	16	RCT	14/16	11/16	NA	NA	Enema,15 g/150 mL, 2 times/d	NA	72 W
Kun et al.	2019	23	23	RCT	21/23	16/23	NA	NA	Oral, 5 g, 4 times/d	VAS	3 M
Guo et al.	2019	30	30	RCT	27/30	19/30	NA	NA	Oral, 5 g, 4 times/d	NA	3 M
Qu et al.	2021	45	45	RCT	45/47	36/45	NA	NA	Oral, 5 g, 4 times/d	VAS	12 W

Exper, experiment; ADR, Adverse Drug Reactions; NA, not available; W, week; M, month; D, day; DLQI, dermatology life quality index; SF-36, Short Form-36 Health Survey; VAS, visual analog scale; NRS, numeric rating scale; 5-DIS, 5-dimensional itching scale; RCT, randomized controlled trial; PSA, Pruritus Severity Assessment.

Based on the extracted data (Table 1), subgroup analyses were conducted concerning dosage and treatment duration. The majority of the included randomized controlled trials employed a consistent regimen of oral Uremic Clearance Granules at 5 grams, four times daily. Analyses focusing on this standard dosage demonstrated its superior effectiveness in improving pruritus compared to control groups. In terms of treatment duration, which varied across studies, the most robust evidence supports a medium-term course of approximately 12 weeks. While data on long-term efficacy (>3 months) are promising, they are less abundant. Consequently, the most substantiated regimen appears to be 5 g orally, four times daily for 12 weeks. However, definitive comparisons with alternative dosages or administration routes are precluded by the limited number of relevant studies. Therefore, future research should include to comparing different regimens and validate long-term sustained benefits.

Two independent reviewers assessed the risk of bias and reporting quality of the included studies through the Cochrane Collaboration’s evaluation tool, categorizing each criterion as low, unclear, or high risk. Disagreements between reviewers will first be addressed through discussion, with unresolved issues escalated to a third reviewer for arbitration. Each study will be scrutinized across seven domains: randomization methods, allocation concealment, blinding of participants and researchers, blinding of outcome assessors, handling of incomplete data, selective reporting, and other biases. Most studies exhibited low risks in randomization, outcome assessor blinding, data completeness, and reporting transparency, while challenges persisted in allocation concealment and participant blinding. Overall, as summarized in Table 2, most of the included RCTs were of moderate quality.

**Table 2:** Cochrane collaboration’s tool for quality assessment in all included trials.

Trials (Author)	Year	Sequence generation	Allocation concealment	Blinding of outcome assessors	Incomplete outcome data	Selective outcome reporting	Others
Yu et al.	2017	Low	Unclear	Low	Low	Low	Low
Sun et al.	2018	Unclear	Low	Low	Low	Unclear	Unclear
Tan et al.	2018	Low	Unclear	Low	Low	Low	Unclear
Cao et al.	2019	Unclear	Low	Low	Low	Low	Low
Li et al.	2019	Low	Unclear	Low	Low	Low	Low
Xi et al.	2021	Low	Low	Unclear	Low	Low	Unclear
Zhang et al.	2023	Unclear	Low	Low	Low	Low	Low
Yang et al.	2016	Low	Unclear	Low	Low	Low	Low
Chen et al.	2020	Low	Low	Unclear	Low	Low	Low
Sun et al.	2020	Low	Low	Low	Low	Low	Low
Gao et al.	2012	Low	Low	Unclear	Low	Low	Low
Kun et al.	2019	Low	Unclear	Low	Low	Low	Unclear

### Outcomes of meta-analysis

A total of 14 RCTs involving 1,232 patients with uremic pruritus, were included. Detailed results are presented in Table 3 and summarized as follows.

**Table 3:** The outcomes of this meta-analysis.

	Studies numbers	Sample size		Overall effect			Heterogeneity	
		Experiment	Control	Effect estimates	95% CIs	*p*-Value	*I*^2^ (%)	*p*-Value
Visual Analogue Scale	8	407	403	WMD = −2.151	−2.372 to −1.930	***p* < 0.001**	64.2%	*p* = 0.007
5D-itch Scale	2	114	114	WMD = −7.642	−14.042 to −1.243	***p* = 0.019**	99.2%	*p* < 0.001
Effective rate	10	369/421	287/415	RR = 1.249	1.169–1.335	***p* < 0.001**	0.0%	*p* = 0.993
Adverse drug reaction	3	13/174	10/174	RR = 1.303	0.589–2.884	*p* = 0.514	0.0%	*p* = 0.938
Serum calcium	8	382	378	WMD = −0.041	−0.577 to 0.495	*p* = 0.881	99.78%	*p* < 0.001
Serum parathyroid hormone	5	226	222	WMD = −93.026	−141.908 to −44.144	***p* < 0.001**	95.4%	*p* < 0.001
Serum Phosphorus	9	440	436	WMD = −0.290	−0.427 to −0.154	***p* < 0.001**	91.2%	*p* < 0.001
Serum intact parathyroid hormone	4	214	214	WMD = −93.383	−238.012 to 51.246	*p* = 0.206	99.2%	*p* < 0.001
Serum creatinine	6	289	289	WMD = −198.788	−323.314 to −74.262	***p* = 0.002**	99.7%	*p* < 0.001
Blood urea nitrogen	6	289	289	WMD = −7.173	−10.560 to −3.785	***p* < 0.001**	97.2%	*p* < 0.001
Uric acid	4	175	175	WMD = −24.518	−64.240 to 15.204	*p* = 0.226	97.1%	*p* < 0.001
β2-microglobulin	5	289	285	WMD = −9.270	−11.613 to −6.927	***p* < 0.001**	98.2%	*p* < 0.01
High-sensitivity C-reactive protein	7	354	350	WMD = −1.801	−2.539 to −1.063	***p* < 0.001**	92.0%	*p* < 0.001
Tumor necrosis factor-α	2	94	94	WMD = −15.234	−20.002 to −10.465	***p* < 0.001**	6.0%	*p* = 0.302
Interleukin-6	2	94	94	WMD = −6.133	−7.422 to −4.844	***p* < 0.001**	0.0%	*p* = 0.515

CIs, confidence intervals; RR, risk ratio; WMD, weighted mean difference; Bold indicates that *p* is less than 0.05.

### Visual analogue scale and 5D-itch scale

**Figure 2:**
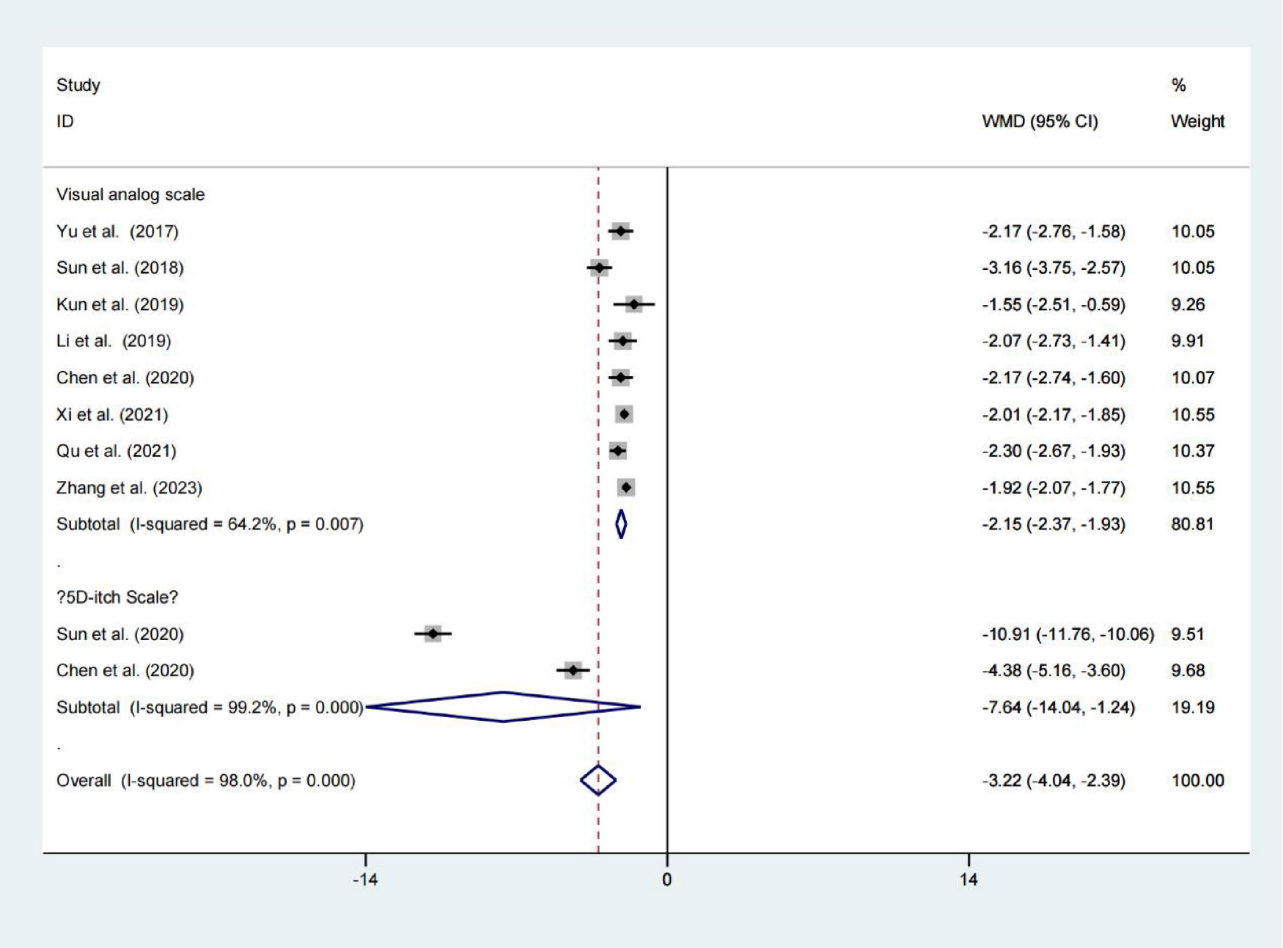
Forest plot for examining visual analogue scale and 5D-itch scale.

Data from eight studies indicated that the Niaoduqing Granule group had significantly lower visual analogue scales than the control group (WMD = −2.151; 95% Cis = −2.372 to −1.930; *p* < 0.001; *I*^2^ = 64.2%; Figure 2). Data from two studies indicated that the Niaoduqing Granule group had significantly lower 5D-itch scale than the control group (WMD = −7.642; 95% Cis = −14.042 to −1.243; *p* = 0.019; *I*^2^ = 99.2%; Figure 2). Notably, a marked attenuation of uremic pruritus severity was achieved in patients following Niaoduqing Granule.

### Effective rate and adverse drug reaction

**Figure 3:**
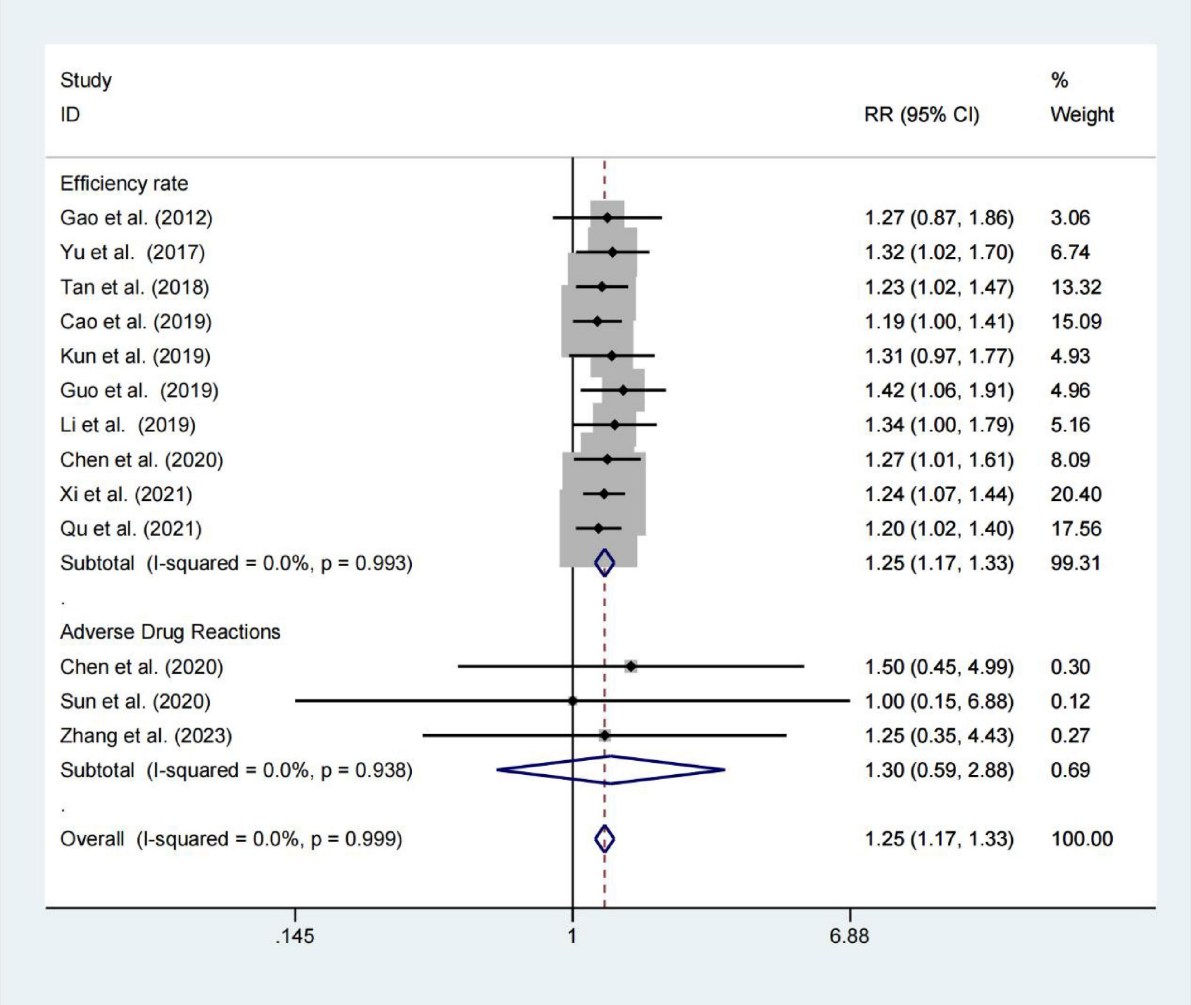
Forest plot for examining effective rate and adverse drug reactions.

Ten studies reported the effective rate, with 421 patients in the experimental groups and 415 patients in the control groups. There was no significant heterogeneity (*p* < 0.001; *I*^2^ = 0.0%), meanwhile, we applied a random-effects model. The results revealed a statistically significant difference between the two groups (RR = 1.249; 95% CIs = 1.169–1.335; *p* < 0.001; Figure 3). Adverse drug reactions were assessed in three trials, showing no significant difference between the Niaoduqing Granule group and control groups (RR = 1.303; 95% CIs = 0.589–2.884; *p* = 0.514; *I*^2^ = 0.0%; Figure 3). Niaoduqing Granules treatment demonstrated significant therapeutic efficacy enhancement in patients, with no concomitant increase in adverse drug reactions, thereby validating the regimen’s safety profile.

### Serum calcium and serum parathyroid hormone

**Figure 4:**
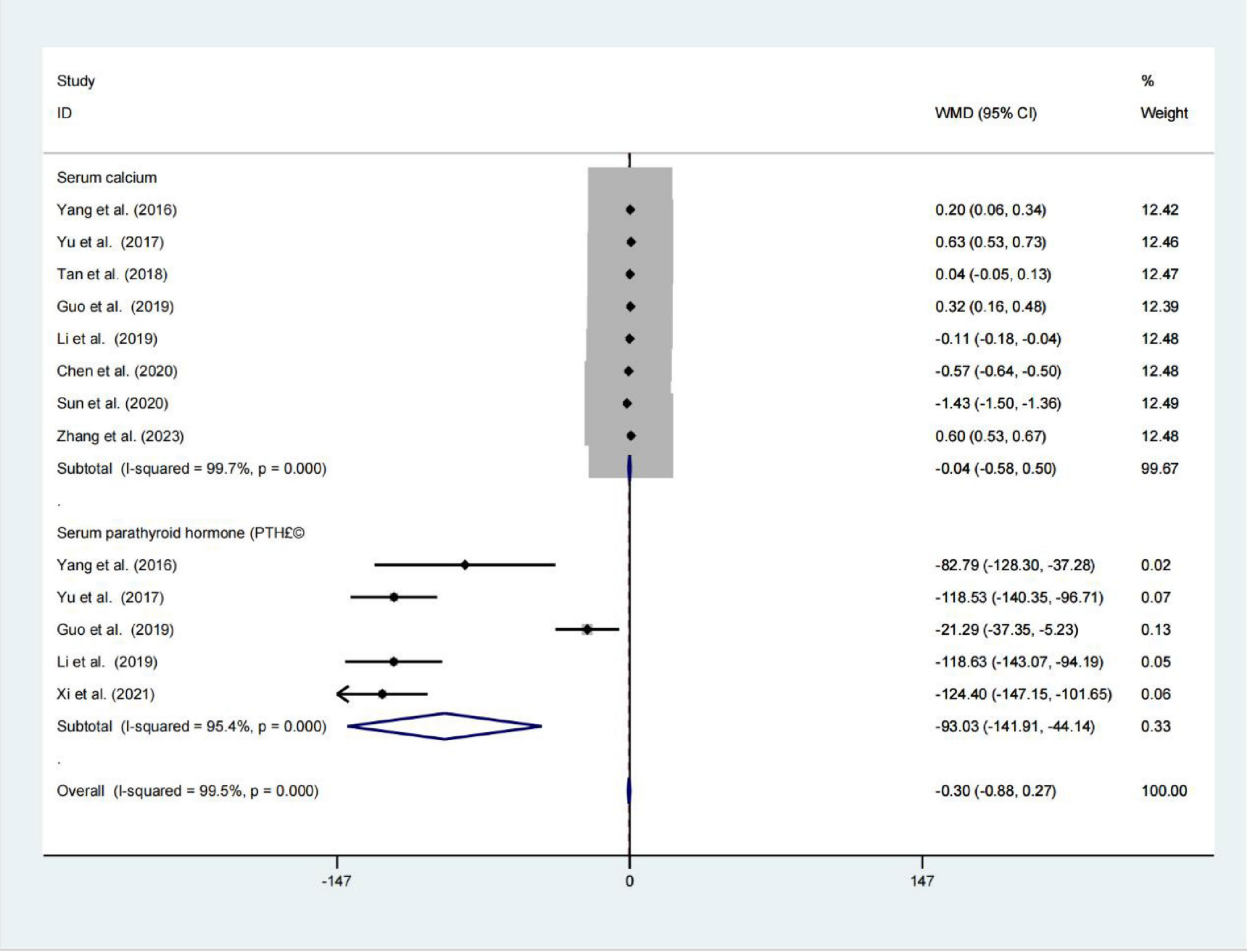
Forest plot for examining serum calcium and serum parathyroid hormone.

Data from eight studies indicated that had no significant difference in serum calcium levels was observed between the two groups (WMD = −0.041; 95% CIs = −0.577 to 0.495; *p* = 0.881; *I*^2^ = 99.78%; Figure 4). Five studies find significant difference serum parathyroid hormone levels was observed between the two groups (WMD = −93.026; 95% CIs = −141.908 to −44.144; *p* < 0.001; Figure 4).

### Serum phosphorus and serum intact parathyroid hormone

**Figure 5:**
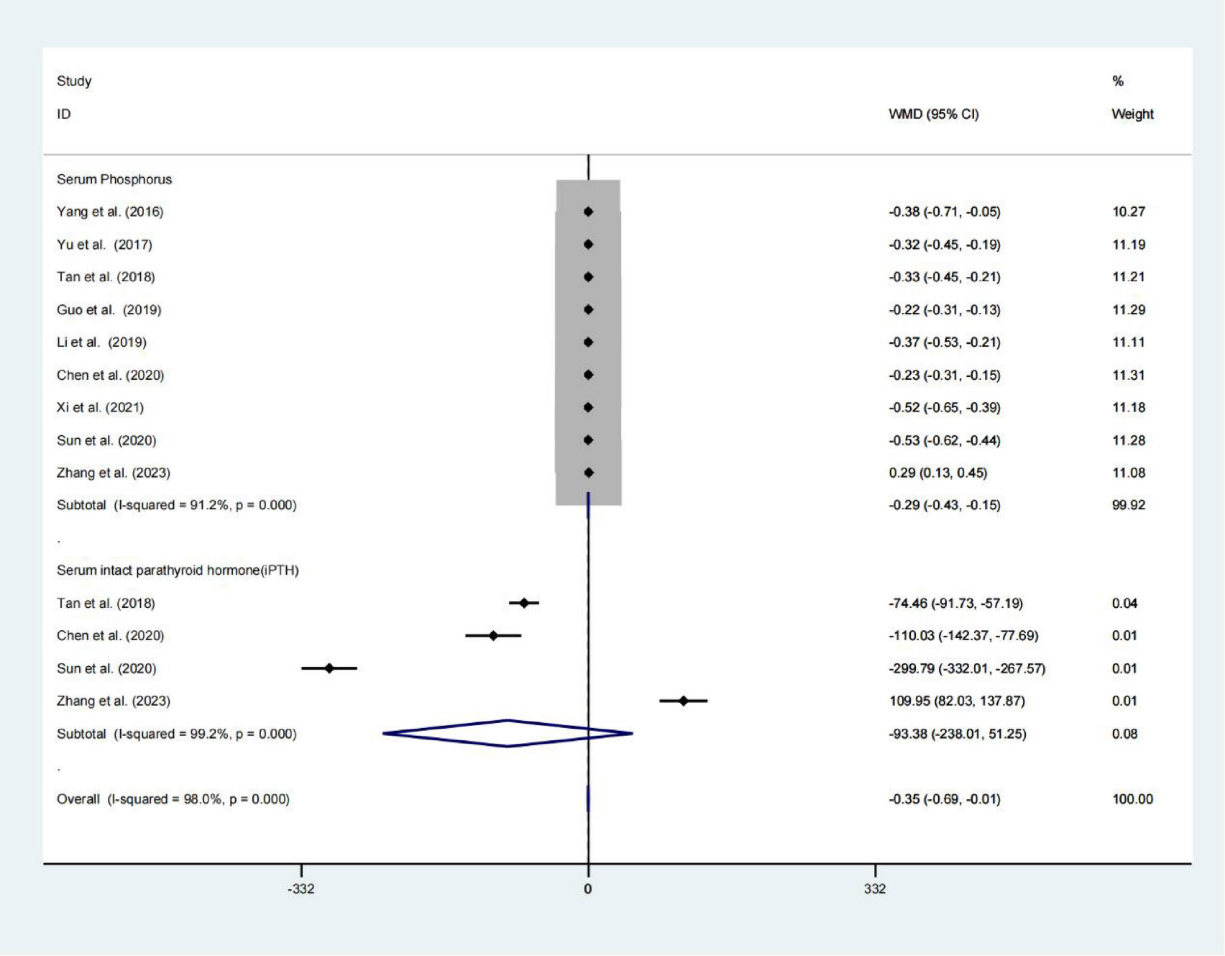
Forest plot for examining serum phosphorus and serum intact parathyroid hormone.

Nine studies found a statistically significant difference in serum phosphorus levels between the two groups (WMD = −0.290; 95% CIs = −0.427 to −0.154; *p* < 0.001; Figure 5). Four analyses do not find a significant difference between the two groups Serum intact parathyroid hormone levels, whereas, a high heterogeneity presents in these studies (WMD = −93.383; 95% CIs = −238.012 to 51.246; *p* = 0.206; *I*^2^ = 99.2%; Figure 5).

### Serum creatinine and blood urea nitrogen

**Figure 6:**
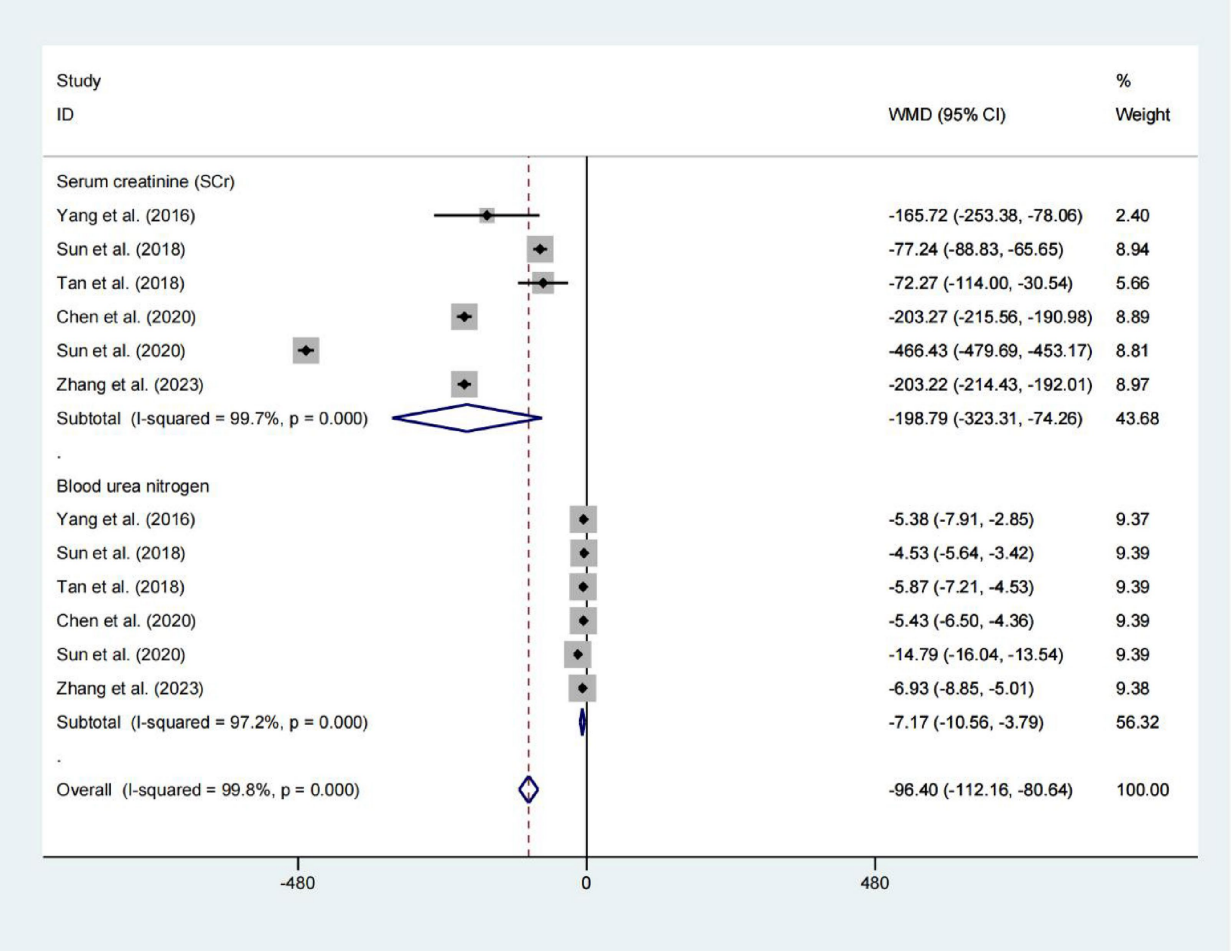
Forest plot for examining serum creatinine and blood urea nitrogen.

A pooled analysis of 578 patients from six studies showed a reduction in Serum creatinine levels (WMD = −198.788) and blood urea nitrogen levels (WMD = −7.173), with *p* values indicating statistical significance (*p* = 0.002 and *p* < 0.001, respectively). However, due to the extremely high heterogeneity (*I*^2^ > 97%), these pooled estimates should be interpreted with extreme caution. The substantial variability among studies precludes drawing reliable conclusions about the effect (Figure 6).

### Uric acid and β2-microglobulin

**Figure 7:**
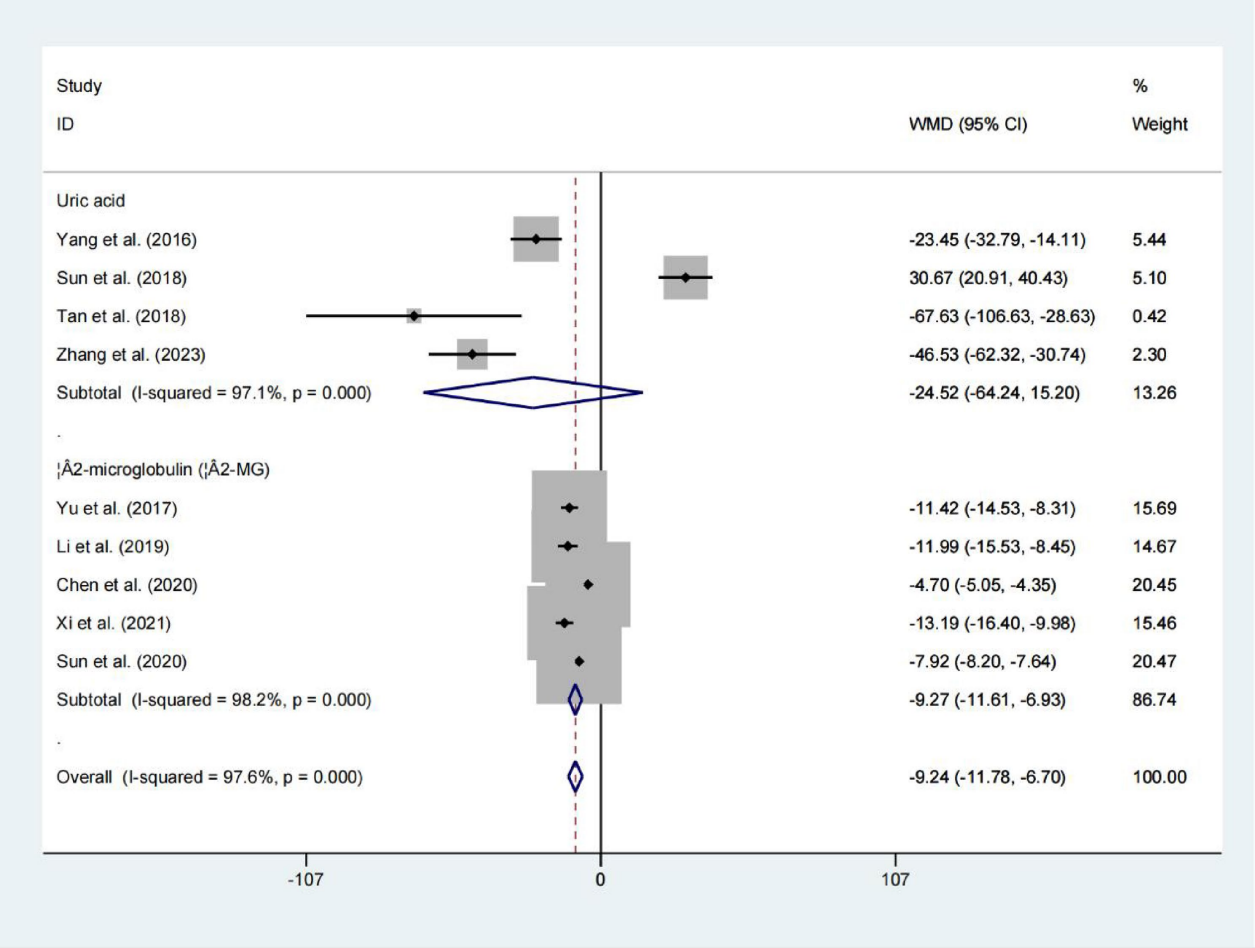
Forest plot for examining uric acid and β2-microglobulin.

Four studies evaluated serum Uric acid levels, upon pooling the results, no significant difference in uric acid levels was observed between the two groups (WMD = −24.518; 95% CIs = −64.240 to 15.204; *p* = 0.226; Figure 7). However, the results showed that the levels of β2-microglobulin in the two groups differed statistically significantly. (WMD = −9.270; 95% CIs = −11.613 to −6.927; *p* < 0.001; Figure 7).

### High-sensitivity C-reactive protein, tumor necrosis factor-α, and Interleukin-6

**Figure 8:**
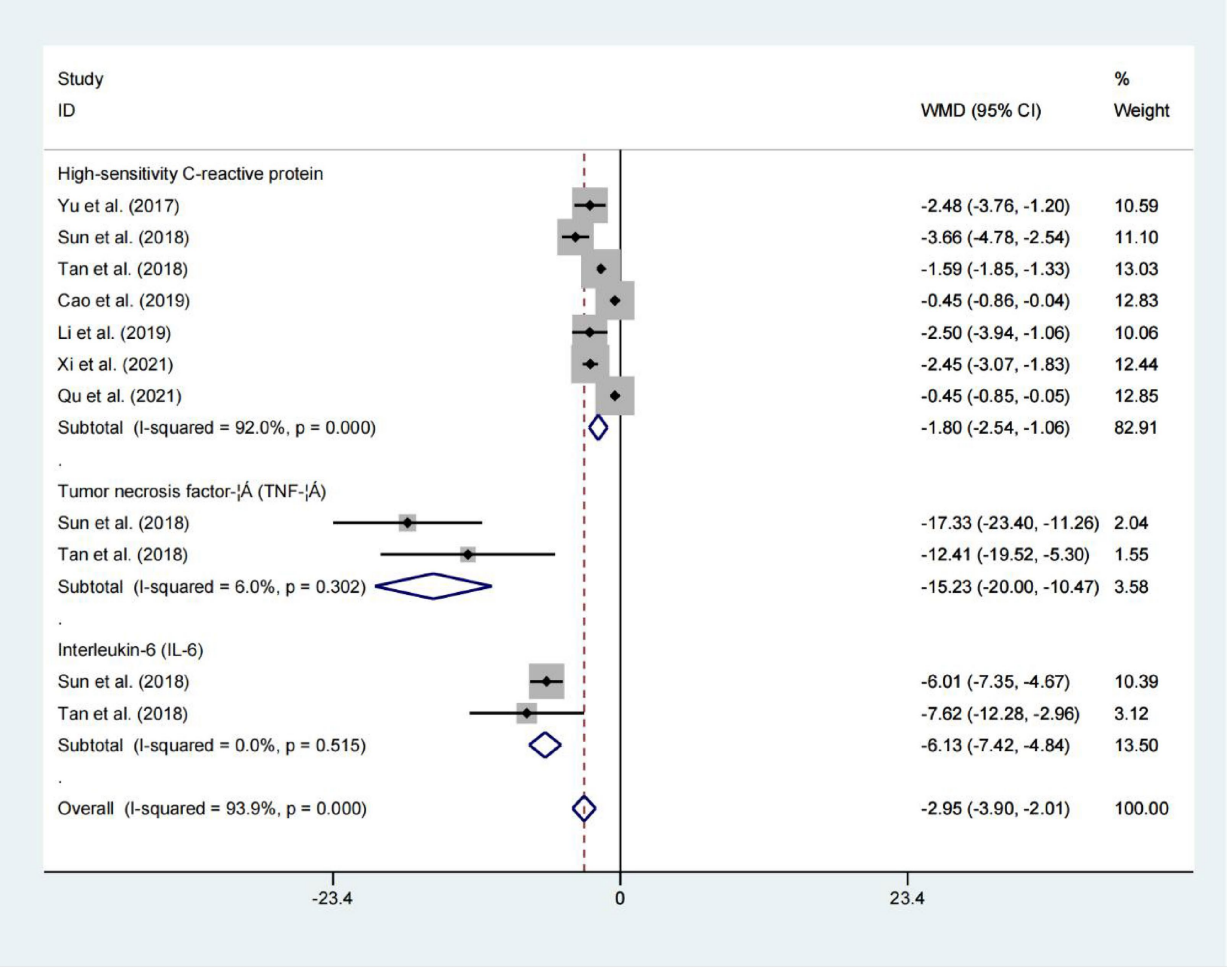
Forest plot for examining inflammatory markers.

Data from seven studies indicated that high-sensitivity c-reactive protein, tumor necrosis factor-α and interleukin-6 levels all demonstrated statistically significant difference between the two groups. As summarized in Figure 8 (High-sensitivity C-reactive protein: WMD = −1.801; 95% CIs = −2.539 to −1.063; *p* < 0.001; Tumor necrosis factor-α: WMD = −15.234; 95% CIs = −20.002 to −10.465; *p* < 0.001; Interleukin-6: WMD = −6.133; 95% CIs = −7.422 to −4.844; *p* < 0.001), whereas, there was no significant heterogeneityininterleukin-6 levels and tumor necrosis factor-α levels. Demonstrates efficacy in attenuating inflammatory burden among hemodialysis patients.

## Discussion

Niaoduqing Granules, a canonical traditional Chinese medicine formula, composed of *Rheum palmatum* (rhubarb), Astragalus membranaceus, *Morus alba* (mulberry bark), *Sophora flavescens*, and Poria cocos (9, 14, 26, 27). These ingredients work together to target the buildup of uremic toxins and metabolic abnormalities in individuals with chronic kidney disease by exerting detoxifying, diuretic, anti-inflammatory, and antioxidant actions. Accumulating evidence supports its adjunctive efficacy in mitigating uremic pruritus via multimodal mechanisms (12–14), a debilitating complication in end-stage renal disease (28). Meta-analytic synthesis of recent clinical trials demonstrates that Niaoduqing Granules, when combined with high-flux hemodialysis (HFHD) or hemoperfusion (HP), significantly reduces pruritus severity and improves clinical response rates compared to dialysis alone (12, 13, 15, 20, 21, 24–26). Mechanistically, Niaoduqing Granules ameliorates biochemical derangements: it lowers serum creatinine and urea nitrogen, while modulating inflammatory mediators. Notably, its antipruritic efficacy correlates with reductions in phosphorus levels and β2-microglobulin (12, 15, 16, 22–25).

The dialysis-specific indicators focused on in this study are of critical clinical significance for a comprehensive assessment of patient outcomes. Dialysis adequacy (serum creatinine, blood urea nitrogen, uric acid, β2-microglobulin) serves as a fundamental prerequisite for efficacy evaluation, as a lack of comparability in baseline levels may directly interfere with the interpretation of improvement in complications. Mineral and bone metabolism parameters (serum calcium, Serum Phosphorus, Serum intact parathyroid hormone etc.) represent a core pathophysiological component of uremic pruritus, and their changes can directly reflect the therapeutic intervention on the underlying etiology. Standardized pruritus assessment tools (Visual Analogue Scale, 5D-itch scale) are essential for quantifying subjective symptoms, significantly enhancing the scientific rigor and comparability of outcome measures. Meanwhile, inflammatory and nutritional markers (High-sensitivity C-reactive protein) provide objective laboratory evidence for exploring the mechanisms of drug action, such as anti-inflammatory effects. Therefore, a systematic evaluation of these indicators helps to elucidate the comprehensive impact of interventions on hemodialysis patients from multiple dimensions, strengthening the clinical value and academic depth of the study’s conclusions.

This study primarily analyzed the effectiveness of Niaoduqing Granules in treating uremic pruritus among hemodialysis patients. The use of Niaoduqing Granules in hemodialysis patients can effectively alleviate uremic pruritus symptoms and reduce scores on relevant pruritus assessment scales. Chen et al. (20) have conducted research in this area as well. The results demonstrate that, the combination of Niaoduqing Granules with high-flux hemodialysis effectively improves scores on the 5-D Pruritus Scale, Visual Analog Scale, and Dermatology Life Quality Index. Simonsen et al. (29), utilizing validated pruritus intensity measurement tools, have further confirmed the efficacy of Niaoduqing Granules. This demonstrates that combination therapy in hemodialysis patients not only alleviates clinical pruritus symptoms but also enhances their quality of life, with a low incidence of adverse reactions. This finding is also consistent with the research conducted by Xi et al. (15). Niaoduqing Granules exhibit therapeutic effects of removing turbid dampness through bowel regulation, strengthening the spleen and draining dampness, and activating blood circulation to resolve stasis, demonstrating efficacy in treating chronic renal insufficiency (14). The granules contain multiple bioactive compounds, including emodin, isoflavones, and paeoniflorin, which improve renal function through multi-pathway mechanisms. Additionally, they ameliorate microinflammatory states, renal anemia, calcium-phosphorus metabolism disorders, and lipid metabolism dysregulation (30), thereby alleviating uremic pruritus.

The pathogenesis of uremic pruritus involves complex and poorly understood pathophysiological processes. Emerging research suggests that dysregulated immune-inflammatory networks may contribute to the pathogenesis of uremic pruritus. Compared to non-pruritic patients, those with uremic pruritus exhibit significantly higher serum levels of interleukin-6, T-helper 1 cells, C-reactive protein, and leukocyte counts, indicating a systemic proinflammatory state (31). The immune dysregulation theory posits that cutaneous and systemic microinflammatory states may act as pruritogenic drivers, directly contributing to the onset and persistence of pruritus (32). Research has demonstrated that chronic renal failure represents a systemic chronic inflammatory state driven by cytokines and characterized by pro-oxidative processes (8). Domestic scholars have isolated and extracted a variety of effective bioactive (6). Furthermore, studies have identified that herbal constituents such as rhubarb (*Rheum palmatum*), licorice (Glycyrrhiza uralensis), and poria (Poria cocos) exhibit antioxidant, anti-inflammatory, and antiviral properties, mediated by their bioactive compounds and mechanisms of action (14). In uremic patients, significantly elevated levels of serum phosphorus and calcium, hyperparathyroidism, abnormal secretion of sweat and sebaceous glands, and inflammatory responses contribute to the accumulation of toxins and metabolic waste. A portion of these toxins are excreted through sweat, leading to the formation of crystals on the skin surface, which irritate the epidermis and trigger intense pruritus. Concurrently, the buildup of toxins (32) and waste products (e.g., β2-microglobulin) that cannot be adequately cleared by the kidneys results in abnormal serum biomarkers, further exacerbating pruritus. Notably, the incidence of pruritus increases with prolonged hemodialysis duration.

Niaoduqing Granules have been used extensively for over 20 years to treat chronic renal disease. They were created using the principles of traditional Chinese medicine (7). According to studies, Niaoduqing Granules, when used as an adjuvant therapy, are more effective than conventional therapy alone. They improve renal function, glucose and lipid metabolism, clinical outcomes, and oxidative stress and inflammatory indicators (4). For uremic pruritus, conventional hemodialysis alone often yields suboptimal therapeutic outcomes. However, adjusting dialysis modalities (e.g., high-flux hemodialysis or extended dialysis sessions) and combining them with Niaoduqing Granules have demonstrated superior efficacy (24). Clinical studies report significant alleviation of pruritus, improvement in renal function (e.g., reduced serum creatinine and urea nitrogen), normalization of biochemical parameters (e.g., calcium-phosphorus balance), and enhanced quality of life in patients receiving this integrated approach (27).

Notably, several limitations warrant consideration to our current study. First off, all of the studies we included are randomized controlled trials, though exclusively conducted in Chinese populations, which places restrictions on their geographic scope. The generalizability of the findings is limited by the single ethnicity of the study population. Furthermore, an optimal dosing strategy remains to be established due to the lack of dose–response investigations. The sample sizes are also somewhat small, ranging from a minimum of 32 to a maximum of 128 people, the observation period was relatively short, ranging from 4 weeks to 72 weeks. Despite the fact that we included 14 research. Lastly, the lack of a gold standard resulted in substantial variability in several outcomes; additional thorough and well-designed research is necessary to reach a more conclusive conclusion. To further confirm Niaoduqing Granules’ mechanism of action in uremic pruritus in hemodialysis patients, higher-quality research is required. The dose-effect relationship for its anti-inflammatory and antipruritic actions must be established through multi-center validation. Long-term monitoring is also necessary to elucidate its effects on complications and productivity.

## Conclusion

Overall, this study confirms the high prevalence of uremic pruritus in hemodialysis patients and its negative impact on quality of life, underscoring the need for effective treatments. Our analysis reveals that Niaoduqing Granules significantly reduce pruritus severity, improving symptoms with a favorable safety profile. These findings support its role as an adjunct therapy. Further large-scale trials are needed to optimize dosing, confirm long-term efficacy, explore mechanisms linking pruritus relief and mechanisms of resistance.

## Data Availability

The original contributions presented in the study are included in the article/supplementary material, further inquiries can be directed to the corresponding author.
